# Radial distraction may reduce the incidence of ulnar-sided wrist pain in ulna-plus morphology intraoperatively following distal radius fractures fixation

**DOI:** 10.1186/s12891-022-05525-0

**Published:** 2022-06-15

**Authors:** Hsuan-Hsiao Ma, Hui-Kuang Huang, Cheng-Yu Yin, Yi-Chao Huang, Ming-Chau Chang, Jung-Pan Wang

**Affiliations:** 1 Department of Orthopedics and Traumatology, Taipei Veterans General Hospital, No. 201, Sec 2, Shi-Pai Road, Taipei, 112 Taiwan, ROC; 2 Department of Orthopedics, School of Medicine, National Yang-Ming Chiao Tung University, Taipei, Taiwan; 3 Division of Orthopaedics, Department of Surgery, Taipei Veterans General Hospital Taitung Branch, Taitung, Taiwan; 4 Department of Orthopedics, Chia-Yi Christian Hospital, Chiayi, Taiwan; 5 Chung Hwa University of Medical Technology, Tainan, Taiwan

**Keywords:** Distal radius fracture, Radial distraction, Ulnar-sided wrist pain

## Abstract

**Introduction:**

Fixed-angle plate fixation can be an effective treatment for distal radius fractures (DRFs). However, patients with existing ulnar positive variance might be at risk of developing symptoms of ulnar-sided wrist pain (USWP). Ulnar shortening osteotomy (USO) is one of the main treatment options for USWP. We hypothesized that a limited radial distraction at the fracture site at the time of surgery for DRF would be functionally equivalent to an indirect USO and that if this were done in a patient with an ulnar plus morphology it could potentially decrease the risk of USWP.

**Methods:**

This retrospective study was conducted at a single institution and all the surgeries were performed by single surgeon. A total of 136 patients (92 women and 44 men) with a mean age of 55 years were enrolled with 57 patients in the distraction group (from 2014 to 2017) and 79 patients (from 2011 to 2013) in the non-distraction group. Patients were assessed USWP. Functional outcomes were assessed using the Disabilities of the Arm, Shoulder and Hand (DASH) questionnaire, Visual Analogue Scale (VAS) for pain, grip strength, and range of motion for the wrist.

**Results:**

The mean follow-up was 37.9 months (range, 28–61 months). The radiographs at postoperative 2-year follow-ups showed the mean ulnar positive variance was 1.3 mm (range, 1–2 mm) in the distraction group and 3.5 mm (range, 2-5 mm) in the non-distraction group. The average of the distraction length was 2.32 mm (range, 2–3 mm). At the 2-year follow-ups, USWP presented in 7% (four patients) in the distraction group, which was significantly less than the incidence of 28% (22 patients) in the non-distraction group. The distraction group exhibited significantly better DASH scores and grip strength and less subsequent ulnar-shortening osteotomy for ulnar-sided wrist pain.

**Conclusions:**

The radial distraction procedure performed during DRFs fixation could possibly reduce the occurrence of postoperative USWP and improve the functional outcomes.

**Level of evidence:**

Level III, Therapeutic.

## Introduction

Residual ulnar-sided wrist pain (USWP) is not uncommon following treatment of distal radius fractures (DRFs) [1]. Many of the symptoms that patients with USWP coexisting with the DRFs may be caused by ulnar impaction syndrome [2], lunotriquetral ligament injuries, extensor carpi ulnaris tendinitis and triangular fibrocartilage complex (TFCC) injuries [3]. USWP attributed to an ulnar impaction syndrome and related to an ulnar-plus variance could be even worse after a DRF [4]. Although the volar locking plate fixation is a good choice to treat the DRFs and might maintain the reduction of the fractures, Madsen et al. found that a shortening of the radius could still occur in 9% of the cases immediately following surgery and 22% after 5 weeks [5]. Thus, if radial shortening occurs, the chance of ulnar impaction and then USWP would be higher.

Ulnar shortening osteotomy (USO) is one option for treatment of USWP following distal radius fracture fixation for the aforementioned causes [2, 6, 7]. USO can decrease ulnar impaction after any desired length of ulnar resection and tighten the ulno-carpal ligamentous complex [3]. However, prolonged immobilization, prominent scarring, risk of delayed union or nonunion, and the removal of symptomatic hardware are disadvantages of the sequential USO to solve the USWP after distal radius fixation [2].

The radial distraction technique was proposed by Fufa et al. [8] An indirect ulnar shortening effect by distraction through the fracture site of the distal radius might be able to address the distal radioulnar joint instability during the volar plating. Theoretically, the distraction technique may also increase the radial length and decrease the incidence of USWP.

The aim of this study was to compare the occurrence of USWP and functional outcome in minimal 2-year follow-up after using radial distraction method or not in ulna-plus morphology intraoperatively following distal radius fracture fixation.

## Materials and methods

This retrospective study was conducted at a single institution and the protocol of this study was approved by the ethics committee of the institution. We included patients whose operation was performed by a single hand surgeon.

**Fig. 1:**
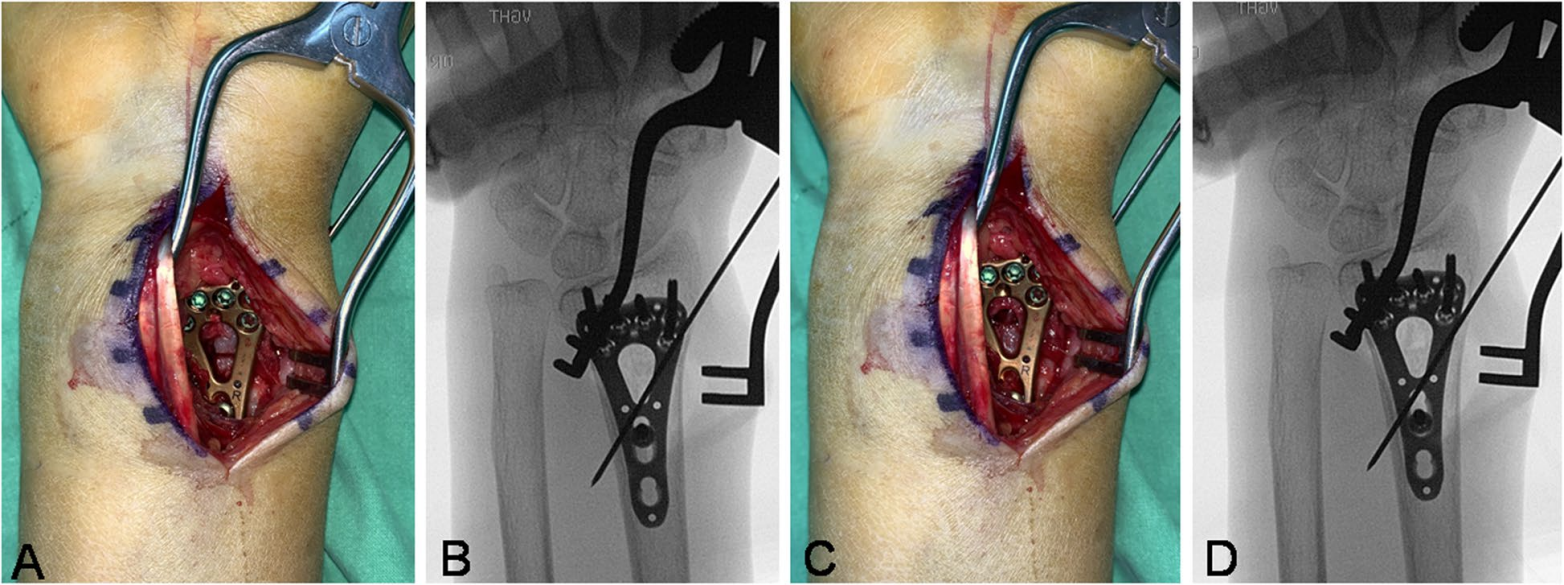
Demonstrating the indirect ulnar shortening after radial distraction via radiographs and photographs. **A** Before distraction, the volar cortex was contact, **B** Before distraction, the ulna-plus morphology was found in the intraoperative radiograph after reduction, **C** After distraction, the gap of the volar cortex was found, **D** After distraction, the positive ulnar variance became smaller than before distraction

Since 2014, we started to adapt the distal distraction method for patients with ulnar plus variance which were noticed during the operation just after the DRFs were reduced and initially fixed (Fig. 1A-D). We reviewed patients who had DRFs and were treated with volar locking plate fixation. From 2014 to 2017, we enrolled patients who underwent the “distraction method” only for the reason of ulnar plus, noted during the initial fracture reduction and still displaying ulnar positive variance intraoperatively. We also enrolled the patients from 2011 to 2013 who did not undergo radial distraction and still displayed ulnar plus variance in the intraoperative radiograph.

Patients were excluded who had surgery more than 14 days after the injury, the presence of a physis at the distal radius, deformities or previous surgeries at the same wrist, other fractures of the same upper extremity, scapholunate ligament injury, and postoperative DRUJ instability. In addition, patients who had pre-existing USWP of the operative wrist and who could not have full 2-year follow-ups were excluded.

All radiographs were evaluated by two hand surgeons not involved in the study. The fractures were classified according to the Müller AO classification system [9]. Demographic data were collected from the medical records.

### Surgical technique

All surgeries were performed under general anesthesia with pneumatic tourniquet control. The volar modified Henry approach with the pronator quadratus elevation method was performed. All fractures were treated and fixed with a volar locking plate (Two-Column VA-LCP; Synthes, Oberdorf, Switzerland). We detected the ulnar plus variance based on the intraoperative fluoroscopy. The proximal cortical screw in the oblong hole was purchased first and then the distal locking screws were purchased. In the distraction group, the locking screws for the metaphysis purchase were set first and the limb of the plate was fixed with one cortical screw set in the distal portion of the oblong hole. The distraction through the fracture site was performed by loosening the screw in the oblong hole and translating the plate distally using the tip of the Halstead-mosquito forceps. Then, the cortical screw in the oblong hole was re-tightened and the remaining diaphyseal screw holes were secured with locking screws. We determined the distraction length by pushing the plate toward distally to a firm end point, which would be the need to tense the injured or laxed ligaments of the DRUJ. The distraction length is assessed for the migration of the compression screw in the oblong hole of the plate before and after the distraction procedure. The distraction length was recorded at the surgical records (Fig. 1A-D). The bone graft or cement wasn’t used in the enrolled cohort.

### Postoperative management

Follow-up was arranged once every 2 weeks in the first month after discharge and once every month thereafter until 6 weeks after the fracture healing was confirmed. Then, follow-up at every 6-month was arranged and additional visits were arranged if needed.

Following surgery, rehabilitation of the active range of motion for the elbow and digits began immediately and the wrist was immobilized in a below-elbow, volar resting orthosis. The stitches were removed at 2 weeks postoperatively and the wrists were then placed in a removable wrist brace with active range of motion of the wrist being initiated. The brace was discarded at 4 weeks postoperatively. Return to full strength for sport and work was allowed at 12 weeks postoperatively with confirmation of fracture healing of the trabecular bridging across the fracture site on the radiographs.

### Functional evaluation

Postoperative USWP was considered as the primary outcome in our study. For USWP assessment, patients were asked whether they had experienced discomfort or pain on the ulnar side of the wrist during the previous week at the minimum two-year postoperative follow-up. We explained the specific activities known to be associated with USWP to aid patient recollection. These specific activities were: pushing oneself up from a bed or the ground using the injured wrist; lifting a fairly heavy object; and turning a doorknob or steering wheel. If a patient answered ‘yes’ to the question, an ulnocarpal stress test was done subsequently to the patient: axial stress was applied to the maximally ulnar deviated wrist and the wrist was then brought through pronation and supination as a provocation [10]. If this maneuver reproduced pain in the injured wrist, we considered it confirmed the presence of USWP. The assessors were blinded to which group the patient was in.

The patient-reported outcome measurement was performed using the Visual Analog Score (VAS) for pain (0, no pain; 10, worst pain) when doing the described aforementioned activities (turning a door knob or steering wheel, lifting a fairly heavy object…) and the Disabilities of the Arm, Shoulder and Hand (DASH) score [11]. We utilized the DASH score as patient-reported outcome (PRO) given as the elbow function may be affected after the ulnar length changing. Grip and pinch strength were measured by a Jamar dynamometer (Sammons Preston, Bolingbrook, IL, USA) which recorded as percentages of the uninjured side. Range of motion was measured using a goniometer and was presented in degrees. Wrist flexion, extension, supination, pronation, radial deviation and ulnar deviation were documented.

### Radiographic evaluation

Posteroanterior (PA) and lateral radiographs of the wrist taken before surgery and 2-years after surgery were evaluated. The radiographs of the contralateral wrist were also evaluated at 2-year after surgery. The radiographic parameters of volar tilt, radial inclination, ulnar variance was evaluated by two independent observers who were blinded to the surgery. Specifically, we measured the ulnar variance in neutral PA views to ensure no changes in ulnar variance in relation to forearm rotation [12]. Follow-up radiographs were also to be evaluated to determine time to radiographic union which defined as bridging callus across the fracture lines at least 2 cortices [13].

### Statistical analysis

The data were represented as mean, range and standard deviation for continuous variables, or the number and percentages for categorical variables. All data were entered and analyzed with SPSS software (version 17.0, SPSS Inc., Chicago, IL). Fisher’s exact test and paired t-test were used to compare the differences between the two groups at two-year follow-up. Univariate logistic regression analysis was performed to identify the factors such as age, gender, with distraction method, fracture pattern, ulnar styloid fracture, and final ulnar variance affecting postoperative USWP. Forward variable selection method was employed to choose the ‘best’ model, with the significance test for a risk factor entering and remaining at the significance level of 0.05. A *p*-value < 0.05 was considered to be statistically significant. Post-hoc analysis was performed to determine the power of this retrospective study. Under α error probability being 0.05 with the proportion of USWP in each group, power being 0.8 will achieve the adequate sample number enrolled in the study.

## Results

**Fig. 2:**
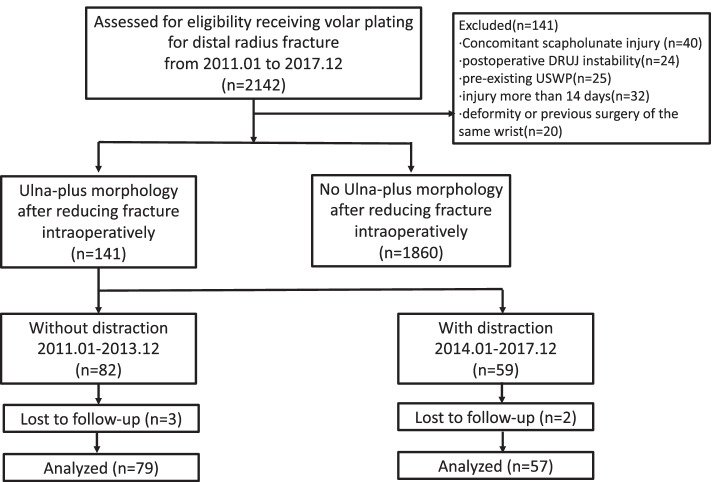
Consort diagram

We reviewed the charts of 2142 patients who were treated by volar plating for distal radius fracture. After adjusting for the patient with exclusion, there were 1860 patients who had no postoperative ulna-plus morphology and 136 patients who met the inclusion criteria and were analyzed (Fig. 2). There were no significant differences in age, gender and fracture classification and union time between two groups. The USWP incidence in the 2-year follow-up was significantly lower in the distraction group (*n* = 4, (7%)) than the non-distraction group (*n* = 22, (28%)). (Table 1).

**Table 1:** Demographic data

	Overall(*N* = 136)	Distraction group(*n* = 57)	Nondistraction group(*n* = 79)	*p*-value
Age	63.4 ± 15.2 (20–84)	64.2 ± 13.5 (20–82)	62.8 ± 16.8 (24–84)	0.786
Sex (Female), n (%)	92(67%)	38(66%)	54(68%)	0.854
Laterality, Right	82(60.3%)	31(54%)	51(65%)	0.308
Dominant hand	32(23%)	12(21%)	20(25%)	0.648
Classification				0.795
AO-OTA 23-A	41(30.1%)	16(28%)	25(31%)	
AO-OTA 23-C	95(69.9%)	41(72%)	54(69%)	
USF, n (%)	91(67%)	39 (68%)	52 (65%)	0.448
Ulnar-sided wrist pain (n, %)	26(19%)	4(7%)	22(28%)	0.002
Union time, weeks	7.8 (5–14)	7.6(6–14)	7.9(5–12)	0.314

*USF* ulnar styloid fracture

Postoperative radiographs 2-year follow-ups showed the mean ulnar positive variance was 1.3 mm (range, 1–2 mm) in the distraction group and 3.5 mm (range, 2-5 mm) in the non-distraction group (*p* < 0.001); while in the contralateral side, the mean ulnar positive variance was 0.1 mm (range, − 2-2 mm) in the distraction group and 0.2 mm (range, − 2-2 mm) in the non-distraction group (*p* = 0.66). The average of the distraction length which was retrieved from the surgical notes was 2.3 mm (range 2–3 mm). At 2-year follow-up, patients that underwent distraction showed better functional outcome in the DASH score (13.1 ± 3.26 vs. 23.4 ± 10.30, p < 0.001) and VAS for pain (0.1 ± 3.26 vs. 0.9 ± 1.60, p < 0.001). The grip and pinch strength showed better percentage of contralateral unaffected wrist in distraction than the non-distraction group. The average wrist range of motion showed no significant in the flexion (75.2 ± 9.27 vs. 75.3 ± 8.1, *p* = 0.92), extension (68.0 ± 8.61 vs. 68.7 ± 9.34, p = 0.66), supination (73.9 ± 6.27 v.s. 72.9 ± 6.88, *p* = 0.369), pronation (62.7 ± 5.06 vs. 61.3 ± 4.73, *p* = 0.103), and radial deviation (29.4 ± 5.84 vs. 28.7 ± 7.47, *p* = 0.560) in distraction group and non-distraction group, respectively. The ulnar deviation was larger in the distraction group (30.5 ± 5.50 v.s. 23.4 ± 5.00, *p* < 0.001). In addition, the incidence of subsequent ulnar-shortening osteotomy was lower in the distraction than the non-distraction group (0% v.s. 15.1%, *p* = 0.001) (Table 2).

**Table 2:** Postoperative functional outcome and radiographic outcome at 2-year follow-up

	Overall (N = 136)	Distraction (n = 57)	Non-distraction (n = 79)	*p*-value
Range of motion, mean ± SD				
Flexion	97.3 ± 8.62	75.2 ± 9.27	75.3 ± 8.1	0.92
Extension	68.4 ± 9.02	68.0 ± 8.61	68.7 ± 9.34	0.66
Supination	73.9 ± 6.62	73.9 ± 6.27	72.9 ± 6.88	0.369
Pronation	61.2 ± 4.90	62.7 ± 5.06	61.3 ± 4.73	0.103
Radial deviation	29.0 ± 6.82	29.4 ± 5.84	28.7 ± 7.47	0.560
Ulnar deviation	26.4 ± 6.26	30.5 ± 5.50	23.4 ± 5.00	< 0.001
Grip Strength (%)	64.9 ± 8.12	72.5 ± 5.07	59.4 ± 4.72	0.001
Pinch (%)	60.0 ± 5.35	61.8 ± 5.13	58.7 ± 5.18	0.001
DASH score	19.1 ± 9.85	13.1 ± 3.26	23.4 ± 10.30	< 0.001
VAS for pain	0.58 ± 1.31	0.1 ± 0.40	0.9 ± 1.60	< 0.001
Radiograph parameter, mean ± SD				
Radial inclination (Degrees)	19.5 ± 5.08	21.7 ± 4.26	18.0 ± 5.09	< 0.001
Contralateral radial inclination (Degrees)	20.5 ± 3.08	20.3 ± 4.15	20.6 ± 4.28	0.683
Volar tilt (Degrees)	13.1 ± 3.21	13.4 ± 3.26	12.8 ± 3.18	0.320
Contralateral Volar tilt (Degrees)	13.2 ± 2.11	12.9 ± 3.14	12.8 ± 3.15	0.816
Ulnar variance (mm)	1.7 ± 1.41	1.3 ± 0.77	3.5 ± 1.19	< 0.001
Contralateral Ulnar variance (mm)	0.20 ± 1.21	0.12 ± 0.92	0.18 ± 1.20	0.716
Distraction length (mm)	–	2.3 ± 1.18	–	–
Subsequent USO	12 (9.0%)	0(0%)	12 (15.1%)	0.001

*USO* ulnar shortening osteotomy, *VAS* visual analog score

The affecting factors associated with postoperative USWP were detected in the multivariate logistic regression analysis. Distraction was the protective factor to the postoperative USWP (aOR = 0.26, *p* = 0.012) and the final ulnar variance was the risk factor (aOR = 1.58, *p* = 0.014) (Table 3).

**Table 3:** Explanatory variables(s) for USWP in multivariate logistic regression analysis

Explanatory variables	Univariate		*p*-value	Multivariate		*p*-value
	OR	95% CI		OR	95% CI	
Sex						
Female (reference)						
Male	1.23	(0.8–1.5)	0.529			
Age	0.92	(0.7–1.2)	0.332			
Distraction	0.32	(0.1–0.6)	0.026*	0.26	(0.1–0.4)	0.012*
Fracture pattern						
AO-OTA 23-A (reference)						
AO-OTA 23-C	1.56	(0.9–1.7)	0.426			
Ulnar styloid fracture	1.32	(0.7–2.0)	0.926			
Final ulnar variance	2.34	(1.9–2.5)	0.002*	1.58	(1.2–1.7)	0.014*

*OR* odds ratio, *AOR* adjusted odds ratio, *CI* confidence interval, *USWP* ulnar-sided wrist pain

*p < 0.05

**Fig. 3:**
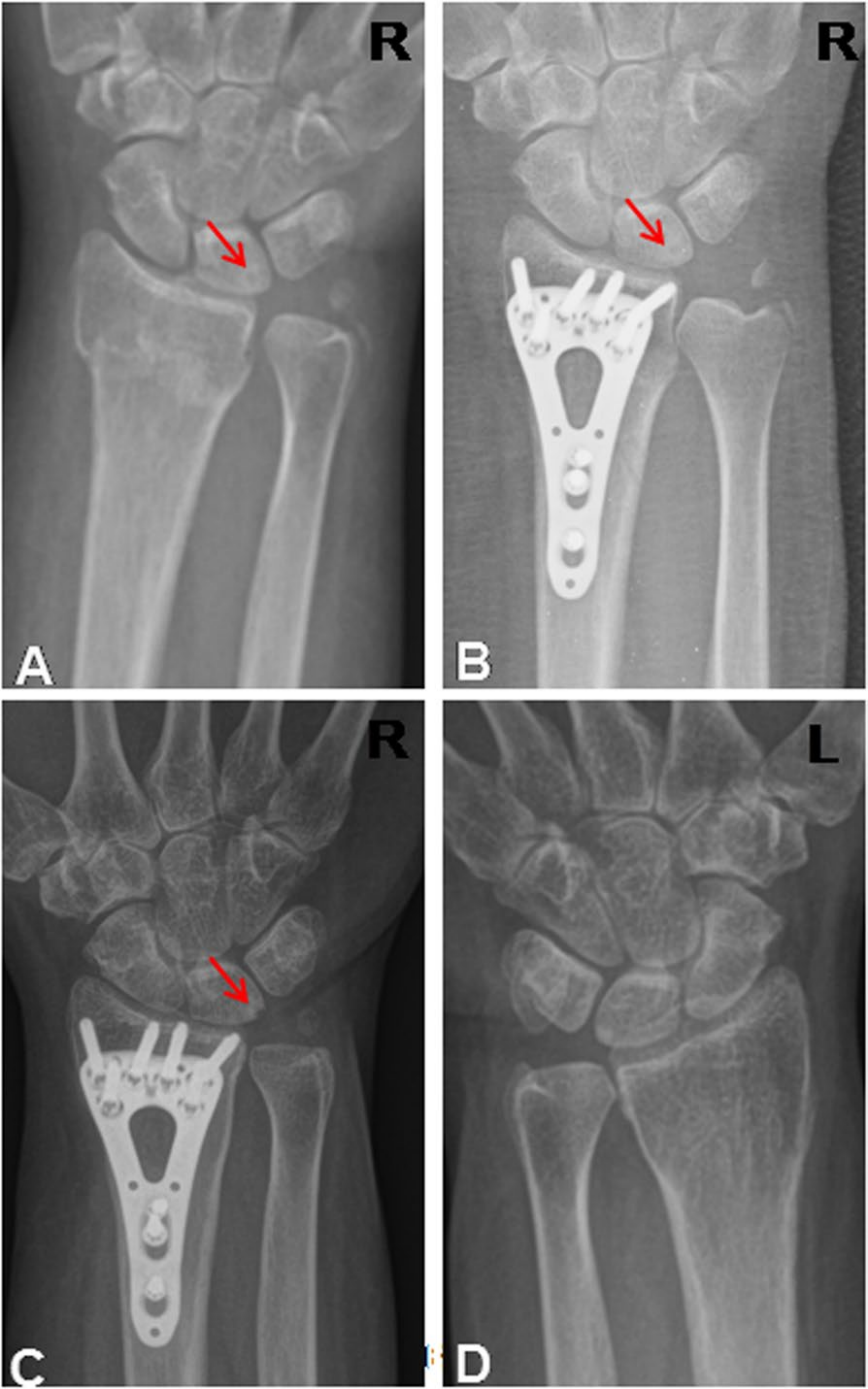
The case demonstrated the subside of the preoperative ulnar-impaction sign on the lunate surface after the distraction surgery. (The red arrow pointed out the sclerotic rim on the lunate surface; R: right, L: left). A: injury film; B: one-month follow-up; C: 24-month follow-up, the sclerotic rim became smaller; D: the contralateral wrist film when 24-month follow-up

Two patients in the distraction group exhibited asymptomatic ulnar-impaction changes on the lunate at the preoperative radiographs. In the follow-ups, the radiographic lesion on the lunate and the lesion became smaller in the serial radiographs (Fig. 3A-D). They didn’t have postoperative USWP at 2-year follow-up. Twelve patients in the non-distraction group had received ulna-shortening osteotomy because of ulna-carpal impingement and this also included five patients with TFCC degenerative tear, and two patients had a lunate-triquetral ligament injury which was found through the arthroscope with the Geissler stage all being stage I with lunate chondromalacia. All displayed improved symptoms in the follow-ups 3 months after the secondary surgery.

There was no nonunion, complex regional pain syndrome, loss reduction requiring return to operative room for revision, infection or any carpal bone avascular necrosis in both groups. In addition, post-hoc analysis showed the measured power under *α* error probability being 0.05 with the proportion of USWP in each group being 0.86.

## Discussion

This study demonstrated the prevalence of the USWP after volar plating to treat DRFs, the prevalence of USWP in patients with ulnar-plus morphology was 7% for patients who had an intraoperative distraction and 28% for patients who did not. The results of this study suggest USWP could affect the functional outcome and that the method of radial distraction is able to decrease the incidence of USWP in patients with ulna-plus morphology following the DRFs fixation.

USWP is highly correlated with a positive ulnar variance. USWP will affect the wrist function. Ulna variance was considered an important prognostic factor such as functional outcome for distal radius fracture. Many reports have shown positive ulnar variance after DRFs healing will impair the wrist function [14, 15]. Our study showed the distraction group had lower positive ulnar variance values and better DASH scores. These findings are in accordance with the results of aforementioned studies. However, there was no data in the literature to confirm the relationship between ulnar variance and other radiographic parameters. In the natural history of the ulna variance in humans, Nathan PA et al. explained that the ulna positive variance configuration was found in about 25% of normal wrists [16]. In addition, some studies found the mean of ulnar variance was positively correlated with age [17, 18]. This study enrolled patients with existing ulna plus variance but who did not complain of USWP before the DRFs. Xu et al. analyzed the severity of ulnar variance change compared with the contralateral wrist on postoperative wrist function in patients with DRFs. The greater severity of radial shortening after DRFs correlated with a poorer grip strength, VAS score, DASH score and wrist range of motion [14]. In our study, radial distraction to have radial lengthening seems to lead to the opposite result in reducing the USWP and achieving better hand function, although the wrist range of motion did not significantly differ. If the USWP happened after the surgery, the reasons may be related to the ulnar plus morphology or others, rising and causing the USWP during the DRFs injuries.

Fufa et al. addressed the radial distraction technique to stabilize the distal radioulnar joint in distal radius fracture [8]. The procedure is unsuitable for AO B-type partial articular fractures due to articular stepping occurring after distraction. In addition, distraction is not indicated for DRUJ deformity or arthritis for worsening DRUJ incongruence which causes pain. The operator may feel a resistant endpoint during the distraction process by using the forceps to push the plate distally. The distraction distance will not be long under this manual method. According to our results, there is no failure of the reduction fixation, no fracture nonunion, no complex regional pain syndrome, and no carpal bone osteonecrosis. In addition, with the limited distance of radial distraction, although the final results were still ulnar-plus variant, the ulnar wrist pain seems able to be decreased. This may imply radial lengthening which is considered as indirect USO may have the same effect as USO.

Isa et al. conducted an in vitro study about the effect of radial lengthening on distal forearm loading. They observed no significant change in the distal radial loads when the radius was lengthened beyond the native length. However, the radial lengthening decreased distal ulnar loading and this may suggest a decrease in ulnar-carpal impaction after the radial lengthening [19]. Their finding is compatible with our study finding.

Gupta et al. reported a biomechanics study concerning the effect of the ulnar-shortening osteotomy being able to reduce the motion between the carpal bones, especially the soft tissue around the lunate-triquetral area [20]. In addition, Mirza et al. demonstrated a clinical study where ulnar-shortening osteotomy could reduce the symptoms of posttraumatic isolated lunotriquetral ligament tears [21]. In our study, two patients of the non-distraction group who had USWP had a lunate-triquetral ligament injury in the arthroscope and received subsequent ulnar-shortening osteotomy. We are uncertain as to whether these finding are associated with the distal radius fracture or the previous lesion before injury. Their symptoms were relieved after USO. As a result, the radial distraction may imply this indirect ulnar-shortening osteotomy could solve the posttraumatic lunotriquetral problem and reduce the USWP. This is consistent with our study. Future studies can investigate the aforementioned relationships.

This study indicated incidences of USWP occurring after the distal radius fracture fixation are not uncommon, and the main causes are ulnar impaction, TFCC injury and lunotriquetral injury. Although there are other likely causes USWP, we need careful evaluation such as provocative maneuvers, image study and diagnostic wrist arthroscopy to identify the cause of USWP [22]. An ulnar shortening osteotomy could be a good choice as the treatment [6, 7, 23]. This radial distraction method is a kind of relative ulnar shortening. We believe when we treat patients with DRFs and we have concern for these problems which will cause existing USWP, then we can perform the radial distraction during the DRF if we choose volar plating as the treatment. This will potentially allow us to treat all these problems at the same radius fixation rather than treat them separately.

In this study, the distraction method was likely to restore the native length of the radius when comparing to the contralateral side of the wrist. However, there is a tendency for metaphyseal distal radius bone to collapse. As a result, the distraction method can achieve an “out to length reduction”, reduce degrees of the ulnar variance positive and decrease the incidence of postoperative USWP.

This study has some inherent limitations. First, the study cohort was from single hand surgeon in one medical center; if future series from multiple surgeons in multiple centers may minimize the inherent bias. Second, it lacks analysis of the AO type-B distal radius fracture, which is not suitable for radial distraction. The wrist arthroscopy evaluations were performed in those patients with USWP, despite conservative treatment (all were in the non-distraction group), and there was no arthroscopy evaluation in the distraction group. In addition, the information of bone quality measurement is not complete in our data collection which may affect the distraction efficacy. Finally, it is impossible to assess the pre-injured condition because of our retrospective study design. To our knowledge, our study presented the functional outcome of radial distraction in ulnar-plus morphology intraoperatively after distal radius fracture fixation. As a result of our adequate sample size through post-hoc analysis, the strength of this study was its ability to make strong clinical conclusions.

In conclusion, this study demonstrated the radial distraction method could reduce the incidence of USWP and achieve a significantly better outcome in patients who still displayed ulna-plus morphology following DRFs fixation. Randomized controlled trials in the future may be recommended.

## Data Availability

The information to access the data used in the study is included within this article.
